# Obesity in the 21st Century: Where Do We Stand?

**DOI:** 10.3390/jcm15208080

**Published:** 2026-10-17

**Authors:** Andrei Modog, Anca Onaciu, Rareș-Mario Borșa, Valentin Toma, Gabriela-Fabiola Știufiuc, Paula Matei, Louise Tedeschi, Cristian Bibu, David Andras, Călin Ionescu, Rareș-Ionuț Știufiuc, Răzvan Scurtu

**Affiliations:** 1 The 6th Surgical Department, “Iuliu Haţieganu” University of Medicine and Pharmacy, Tabacarilor 9-11, 400139 Cluj-Napoca, Romania; modog.andrei@elearn.umfcluj.ro (A.M.); calin.ionescu@umfcluj.ro (C.I.); razvan.scurtu@umfcluj.ro (R.S.); 2 Department of Nanosciences, MEDFUTURE—Institute for Biomedical Research, “Iuliu Haţieganu” University of Medicine and Pharmacy, Louis Pasteur 4-6, 400349 Cluj-Napoca, Romania; anca.onaciu@umfcluj.ro (A.O.); rares.mari.borsa@elearn.umfcluj.ro (R.-M.B.); valentin.toma@umfcluj.ro (V.T.); 3 Department of Pharmaceutical Physics & Biophysics, Faculty of Pharmacy, “Iuliu Haţieganu” University of Medicine and Pharmacy, Louis Pasteur 6, 400349 Cluj-Napoca, Romania; 4 Department of Maxillofacial Surgery and Implantology, Dental Medicine Faculty, “Iuliu Haţieganu” University of Medicine and Pharmacy, Cardinal Iuliu Hossu 37, 400029 Cluj-Napoca, Romania; 5 Department of Prosthetic Dentistry and Dental Materials, Division Dental Propaedeutics, Aesthetics, Dental Medicine Faculty, “Iuliu Haţieganu” University of Medicine and Pharmacy, Clinicilor 32, 400001 Cluj-Napoca, Romania; 6 Faculty of Physics, Babes-Bolyai University, Mihail Kogălniceanu 1, 400084 Cluj-Napoca, Romania; gabriela.stiufiuc@ubbcluj.ro; 7 General Medicine Faculty, “Iuliu Haţieganu” University of Medicine and Pharmacy, Victor Babes 8, 400012 Cluj-Napoca, Romania; matei.paula.cristina@elearn.umfcluj.ro; 8 Department of Surgery, “Iuliu Haţieganu” University of Medicine and Pharmacy, Clinicilor 3-5, 400006 Cluj-Napoca, Romania; louise.mari.tedeschi@elearn.umfcluj.ro (L.T.); cristian.flor.bibumonus@elearn.umfcluj.ro (C.B.); david.andras@umfcluj.ro (D.A.); 9 Nanotechnology Laboratory, TRANSCEND Research Center, Regional Institute of Oncology, General Henri Mathias Berthelot 2-4, 700483 Iaşi, Romania

**Keywords:** precision medicine, obesity diagnosis, obesity treatment, bariatric surgery, biomolecules, multi-omics, analytical methods

## Abstract

Obesity in the 21st century has become a global epidemic owing to its heterogeneous aetiology and clinical presentation, as well as the development of associated comorbidities such as cardiovascular disease, type2 diabetes, hypertension, dyslipidaemia, obstructive sleep apnoea, and obesity-related cancers. Notably, cardiovascular disease and diabetes are major contributors to obesity-associated morbidity and mortality. Current diagnostic approaches rely predominantly on anthropometric measurements, which have proven limited in their ability to provide accurate and clinically meaningful stratification of patients with obesity. Consequently, obesity treatments, including metabolic surgery and pharmacological therapies, remain insufficiently tailored to the wide variability in obesity-related genotypes, phenotypes, and endotypes. In this context, a personalised medical approach offers considerable promise. A clear overview of currently effective diagnostic tools and treatments, as well as future possibilities, is therefore needed. Recent developments in precision medicine, multi-omics, biomarkers, and advanced diagnostic technologies may facilitate improved patient stratification and support more individualized therapeutic strategies. Together, these approaches may contribute to a more comprehensive, evidence-based, and patient-centered framework for the prevention, diagnosis, and management of obesity and its complications.

## 1. Introduction

Obesity in the 21st century has become a silent epidemic owing to a heterogeneous array of factors, among which socioeconomic and environmental determinants, aetiology, clinical presentation, and associated comorbidities are particularly prominent.

Over the past four decades, obesity has evolved from a regional health concern into a full-blown global pandemic. The World Health Organization (WHO), The Lancet, and other key scientific bodies have reported a dramatic and continuous rise in global overweight and obesity rates, affecting both adults and children [1].

Between 1990 and 2021, global obesity prevalence increased by 155% in men and 105% in women. The steepest increases were seen in North Africa and the Middle East, where prevalence more than tripled in men and more than doubled in women [1]. The World Obesity Atlas published in The Lancet in 2025 reported that, according to current trends, by 2030 more than 2.9 billion adults will have an increased body mass index (BMI), of whom 1.1 billion (487 million men and 643 million women) will have clinical obesity (BMI ≥ 30 kg/m^2^) [1].

The current clinical approach is based on anthropometric measurements such as BMI, waist circumference (WC), waist-to-height ratio (WHtR), and waist-to-hip ratio (WHR), as well as clinical scores such as the Edmonton Obesity Score System (EOSS) [2]. Following this diagnostic approach, obesity treatment is generally based on lifestyle modification, with or without pharmacological intervention, and, when indicated, bariatric surgery. Although widely used, this approach has yielded limited population-level results, as reflected by the continuing expansion of the obesity epidemic.

Conventional diagnosis can be complemented by circulating biomarkers reflecting metabolic and inflammatory disturbances, including glucose, insulin, lipid fractions, adipokines, and inflammatory mediators [3]. This field is converging towards personalised medicine, which seeks to tailor diagnosis and treatment to the individual’s biological and clinical profile by integrating precision-medicine frameworks that emphasise patient stratification, prediction of disease trajectories, treatment response, and prevention of relapse [4]. The development of these frameworks has increasingly relied on the growing impact of multi-omics approaches, including genomics, epigenomics, transcriptomics, proteomics, metabolomics, and metagenomics, in both research and clinical settings. This impact has been further amplified by advances in machine-learning capabilities, which facilitate the processing and integration of large and complex datasets.

Considering this evolving paradigm, this review aims to evaluate the available diagnostic and treatment options for patients with obesity and to introduce new perspectives on how obesity management may change in the era of personalised medicine.

## 2. Methods

To provide a comprehensive and balanced overview of obesity in the 21st century, a structured literature search was conducted. Given the broad scope and integrative nature of the present manuscript, a narrative review approach was adopted. Electronic searches were performed using PubMed, Web of Science, and Scopus. The search primarily focused on the literature published between 2000 and 2026 to capture major developments in obesity research during the 21st century. Earlier publications, dating back to 1980, were additionally considered when relevant to provide historical context regarding the evolution of the obesity epidemic and the development of diagnostic and therapeutic approaches. The final literature search was updated in September 2026.

The search strategy combined Medical Subject Headings (MeSH) terms, where applicable, with free-text keywords. The main search combinations included: (“obesity” OR “overweight”) AND (“diagnosis” OR “body mass index” OR “waist circumference” OR “EOSS”); (“obesity” OR “overweight”) AND (“treatment” OR “diet” OR “pharmacotherapy” OR “bariatric surgery”); (“obesity” OR “overweight”) AND (“molecules” OR “biomarkers”); (“obesity” OR “overweight”) AND (“analytical methods” OR “nanotechnology”); (“obesity” OR “overweight”) AND (“personalized medicine” OR “precision medicine”); and (“obesity” OR “overweight”) AND (“omics” OR “multi-omics”).

The literature considered for inclusion comprised peer-reviewed original research articles, randomised controlled trials, systematic reviews, narrative reviews, and meta-analyses published in English, together with relevant reference works when necessary for historical or conceptual context. Studies were considered eligible when they directly addressed the epidemiology, diagnosis, treatment, molecular mechanisms, biomarkers, or advanced analytical and multi-omics approaches related to obesity.

Titles and abstracts were subsequently screened by the authors for relevance to the objectives and scope of the review. Full-text articles considered potentially eligible were then retrieved and assessed in greater detail. Studies were excluded when they provided redundant information, lacked sufficient methodological clarity, or were not directly relevant to the topics addressed in this review. When several publications reported similar findings, preference was given to more recent studies, studies with larger populations, and those providing more comprehensive methodological or clinical information. Accordingly, a total of 186 studies and relevant reference books were included in the final narrative synthesis.

Given the narrative nature of this review and the substantial methodological and clinical heterogeneity of the included literature, no quantitative data synthesis or pooled effect estimates were performed. Similarly, formal assessments of between-study heterogeneity and publication bias were not undertaken. Instead, the available evidence was synthesized narratively, with emphasis on the integration of conventional clinical approaches with emerging molecular, multi-omics, and precision-medicine strategies in obesity diagnosis and treatment.

## 3. Obesity, How Do We Diagnose It?

The diagnosis of obesity is primarily anthropometric and begins with the calculation of BMI. BMI is the most widely used index for obesity and forms the basis of many approaches to the evaluation and treatment of this condition.

The WHO adopted BMI as the main diagnostic method for obesity in 1998. At present, it is widely used both in clinical practice and in research as a rapid, inexpensive, and simple screening and diagnostic tool [5].

Although BMI has been established as a fundamental tool for identifying overweight and obesity, it also has important limitations. Its predictive capacity is restricted by differences in body composition, as it cannot distinguish between adipose tissue, muscle mass, and fluid retention. Moreover, BMI is limited in assessing the clinical impact of obesity on health status and quality of life [5,6].

Other measurements can be used to assess adiposity distribution more accurately. WC, WHtR, WHR, and EOSS can provide a clearer picture of fat distribution, identify features associated with metabolic syndrome (MetS), and evaluate the overall clinical impact of obesity [7].

Measuring WC is a simple and inexpensive procedure that can be performed in primary care settings; in addition, WHtR and WHR can be calculated [6,7].

A 2026 study including 10,747 adults from the U.S. National Health and Nutrition Examination Survey (NHANES) evaluated the diagnostic performance of conventional anthropometric indices for identifying excess adiposity, using whole-body dual-energy X-ray absorptiometry (DEXA) as a reference method. Based on standard Centres for Disease Control and Prevention (CDC) BMI thresholds, BMI alone demonstrated a sensitivity of 74.6% and a specificity of 82.2% for detecting excess adiposity. In contrast, WC showed a higher sensitivity of 88.5%, although at the expense of lower specificity (66.7%). A similar pattern was observed for WHtR, further supporting the complementary role of central adiposity measures in the anthropometric evaluation of obesity [8].

According to the observations of the 2025 Lancet Diabetes & Endocrinology Commission, obesity can be phenotypically classified as android or central obesity, characterised by a predominance of visceral adiposity, or gynoid obesity, characterised by a predominant distribution of adipose tissue in the hips and buttocks. Each of these patterns is associated with distinct implications for cardiovascular risk and obesity-related comorbidities [7].

Consistent with this concept, BMI should be complemented by additional anthropometric measures reflecting fat distribution, such as WC, WHtR, or WHR, or by direct assessment of body fat when available. Integrating these parameters may provide a more accurate characterisation of the obesity phenotype and improve the assessment of obesity-related metabolic and cardiovascular risk [7,9].

Nevertheless, a comprehensive diagnosis should not be limited to anthropometric measurements. A 2024 study conducted in a Danish population screened for diabetes, sleep apnoea, liver disease, and high blood pressure using both clinical parameters and specific blood tests [10]. The study included 769 individuals aged 18–60 years with a BMI above 30 kg/m^2^ and a comparison group with BMI within the reference range. The screening programme identified substantial proportions of previously undiagnosed obesity-related conditions, including diabetes in 4.2%, insulin resistance in 9.1%, increased liver fat in 68.1%, increased liver stiffness in 17.4%, hypertension or masked hypertension in 19.0%, and sleep apnoea in 25.1%. Notably, the study showed an uneven distribution of these conditions, underlining the need for a more personalised approach [10].

In this regard, MetS is closely associated with obesity and is characterised by a combination of clinical features, including excessive abdominal fat (central obesity, commonly assessed by WC [11]), insulin resistance (IR), which may be detected through fasting blood glucose or glucose tolerance testing [12], endothelial dysfunction, and dyslipidaemia, characterised by elevated triglyceride and low-density lipoprotein (LDL) cholesterol levels together with low high-density lipoprotein (HDL) cholesterol levels [13]. These characteristics significantly increase the risk of cardiovascular disease (CVD) and type 2 diabetes mellitus (T2DM). Hypertension, characterised by elevated systolic and/or diastolic blood pressure, may also occur as an obesity-related complication [14]. Finally, the synergistic action of MetS components promotes pro-inflammatory and pro-thrombotic states, reflected by elevated serum C-reactive protein (CRP) and fibrinogen levels and contributing to cardiovascular risk [15,16].

MetS is usually diagnosed by assessing clinical parameters such as WC, WHR, blood pressure, impaired glucose tolerance, serum triglycerides, and cholesterol levels. Different healthcare organisations use different standards and definitions for MetS.

According to the WHO [17], a diagnosis of MetS requires at least three of the following criteria:

- Presence of glucose intolerance, impaired glucose tolerance (IGT) or diabetes mellitus (DM), and/or IR;
- Raised arterial pressure, i.e., ≥140/90 mm Hg;
- Raised plasma triglycerides (≥150 mg/dL) and/or low HDL-C (<35 mg/dL in men and <39 mg/dL in women);
- Central obesity, i.e., WHR > 0.9 in men and >0.85 in women and/or BMI > 30 kg/m^2^,
- Microalbuminuria, i.e., urinary albumin excretion rate ≥ 20 μg/min or albumin/creatine ratio ≥ 30 μg/mg.

The European Group for the Study of Insulin Resistance (EGIR) stipulates that patients with obesity must meet at least three of the following criteria for the diagnosis of MetS [18]:

- Elevated plasma insulin (>75th percentile);
- Abdominal obesity: WC ≥ 94 cm in men and ≥80 cm in women;
- Hypertension: ≥140/90 mm Hg or on antihypertensive treatment;
- Elevated triglycerides (≥150 mg/dL) and/or reduced HDL-C (<39 mg/dL for both men and women);
- Elevated plasma glucose: impaired fasting glucose (IFG) or IGT, but no diabetes.

According to the International Diabetes Federation (IDF) [16,18], MetS is diagnosed when the patient meets at least three of the following criteria:

- Central obesity (according to predefined standard race and gender-specific waist circumference cut-offs);
- Raised triglycerides: ≥150 mg/dL or history of specific treatment for this lipid abnormality;
- Reduced HDL cholesterol: <40 mg/dL in males and <50 mg/dL in females or history of specific treatment for this lipid abnormality;
- Raised blood pressure: systolic BP ≥ 130 mm Hg or diastolic BP ≥ 85 mm Hg or on treatment for previously diagnosed hypertension;
- Raised fasting plasma glucose: ≥100 mg/dL or previously diagnosed type 2 diabetes mellitus.

The link between obesity, MetS, and CVD is evident in both diagnosis and pathophysiology. The mechanisms through which MetS contributes to CVD are closely related to insulin resistance in skeletal muscle and the liver. This reduces glucose uptake and promotes increased lipolysis in adipose tissue, which, over the long term, contributes to atherosclerosis. High glucose levels also increase the formation of advanced glycosylation end products, promote microvascular lesions, and contribute to other components of MetS [16,19,20].

This combination of factors underlies the close relationship between obesity, MetS, and metabolic dysfunction-associated steatotic liver disease (MASLD), an entity previously known as non-alcoholic fatty liver disease and characterised by hepatic steatosis with potential progression to fibrosis and severe hepatic dysfunction. Patients with obesity have a twofold increased risk of developing MASLD compared with individuals without obesity. Furthermore, MASLD and CVD share several associated metabolic abnormalities, including IR, low-grade systemic inflammation, and oxidative stress, which are also characteristic of patients with obesity. This close association between MetS and MASLD highlights the complexity of the metabolic profile of patients with obesity and emphasises the need for a comprehensive diagnostic assessment based on objective clinical data [21].

To evaluate and classify the clinical impact of excess weight, MetS, and other diseases associated with obesity, A.M. Sharma and R.F. Kushner developed the Edmonton Obesity Score System (EOSS) in 2009 [22].

Clinically, EOSS aims to provide for obesity what the Tumour Nodes and Metastasis (TNM) system does for cancer, or the New York Heart Association Functional (NYHA) classification does for heart failure. It grades obesity according to its clinical and biological impact (Table 1). EOSS considers both direct clinical manifestations (e.g., dyspnoea or fatigue with minimal effort, joint pain, limitation of daily activities) and the presence of comorbidities associated with obesity (e.g., type 2 diabetes, hypertension, obstructive sleep apnoea, anxiety disorders, dyslipidaemia), whether or not they are diagnosed at the time of the initial assessment [22,23].

Although conventional anthropometric and clinical assessments remain essential for the diagnosis and staging of obesity, their integration with additional data, such as metabolites, proteins, genetic information, and gut microbiome profiles, may provide a more comprehensive characterisation of disease heterogeneity and obesity-related complications, supporting more individualised patient stratification and management [25,26].

A study conducted across three US states over a four-year period compared anthropometric indices with metabolomic, proteomic, genetic, and gut microbiome data obtained from patients with obesity and individuals without obesity, using machine-learning-based statistical models. The study showed that BMI derived from multi-omics data may more accurately identify individuals with an unfavourable metabolic profile than classification based solely on anthropometric BMI and may also help predict the response to therapeutic interventions such as behavioural and lifestyle interventions (BLI) [27].

## 4. Established Directions for Molecular Evaluation in the Diagnosis and Management of Obesity and Its Related Complications

Through interactions among the central nervous system, gastrointestinal tract, and endocrine system, these systems form a regulatory triad that plays a key role in the processes governing the body’s nutritional functions.

Therefore, adipose tissue plays a central role, functioning as an active endocrine organ [3]. It secretes a wide range of biomolecules—including hormones, chemokines, lipids, cytokines, proteins, and metabolites—with essential roles in appetite regulation, energy balance, metabolism, and inflammation [25].

Based on their roles in energy homeostasis, many of these biomolecules act as either orexigenic (appetite-stimulating, e.g., ghrelin) or anorexigenic (appetite-suppressing, e.g., leptin) regulators, influencing short- and long-term energy balance [3]. Changes in the levels of these molecules have been linked to obesity-related comorbidities, such as insulin resistance, metabolic disturbances, gastrointestinal disorders, hypertension, and cardiovascular disease [28].

Adipokines are a family of cell-signalling molecules secreted by adipose tissue with important roles in the regulation of appetite, inflammation, immunity, and metabolism [29]. They are key regulators of energy balance and can act as hormones, growth factors, and cytokines [29]. Representative adipokines include leptin, ghrelin, adiponectin, resistin, visfatin, chemerin, and omentin-1.

Leptin and ghrelin play central and opposing roles in appetite regulation. Leptin, secreted mainly by white adipose tissue, acts on hypothalamic nuclei to induce satiety and maintain long-term energy homeostasis [30,31]. Although circulating leptin levels increase proportionally with fat mass, obesity is commonly characterised by leptin resistance, resulting in impaired appetite suppression [32]. In contrast, ghrelin, predominantly produced by gastric PD/D1 cells [33], stimulates hunger and food intake while also influencing lipid storage and adipose tissue expansion [34,35]. In obesity, ghrelin activity is frequently reduced owing to elevated leptin levels and central resistance mechanisms, further disrupting appetite regulation [36,37].

Adiponectin exerts protective metabolic effects by enhancing insulin sensitivity and displaying anti-inflammatory properties [38]. Unlike leptin, adiponectin levels are inversely correlated with adiposity, and reduced concentrations in obesity are associated with insulin resistance, metabolic syndrome, type 2 diabetes, and cardiovascular risk [39,40,41]. Adiponectin resistance, mainly affecting liver and skeletal muscle tissues, is driven by receptor downregulation, impaired intracellular signalling, chronic inflammation, and dietary factors [42,43,44].

Other adipokines, including resistin, visfatin, chemerin, and omentin-1, further modulate metabolic and inflammatory pathways in obesity. Resistin acts as a pro-inflammatory mediator and promotes insulin resistance, with circulating levels strongly correlated with BMI and adipose tissue mass, supporting its role as a biomarker for obesity and cardiovascular risk [45,46,47]. Visfatin has insulin-like and pro-inflammatory effects and is associated with visceral adiposity. However, its clinical relevance remains debated [45,48,49,50,51,52]. Chemerin contributes to adipocyte differentiation, insulin resistance, and chronic inflammation [53,54,55], while omentin-1 displays beneficial metabolic effects by enhancing insulin sensitivity and exerting anti-inflammatory actions [56]. Its reduced expression in obesity exacerbates metabolic dysfunction [57,58].

Glucose homeostasis and energy balance are tightly regulated by the coordinated action of pancreatic and intestinal hormones acting at both peripheral and central levels. Insulin, the principal hypoglycaemic hormone, facilitates glucose uptake in muscle and adipose tissue and contributes to satiety signalling within the central nervous system [59,60,61]. In obesity, the altered secretory profile of adipose tissue interferes with insulin signalling pathways, leading to insulin resistance and compensatory hyperinsulinaemia [62]. This adaptive response enhances adipogenesis, suppresses lipolysis, and promotes further fat accumulation [63], thereby establishing a self-perpetuating cycle of metabolic dysfunction that underlies many obesity-associated comorbidities, including type 2 diabetes mellitus [64,65].

Within this dysregulated metabolic context, intestinal incretin hormones play a critical modulatory role. Glucagon-like peptide-1 (GLP-1), released postprandially, amplifies glucose-dependent insulin secretion, suppresses glucagon release, delays gastric emptying, and activates central satiety pathways [66]. Together with glucose-dependent insulinotropic polypeptide (GIP), GLP-1 mediates the incretin effect, which accounts for a substantial proportion of postprandial insulin secretion [66]. Beyond its insulinotropic action, GLP-1 supports pancreatic β-cell function, enhances peripheral glucose utilisation, and contributes to appetite regulation; however, its physiological efficacy is limited by rapid enzymatic degradation in the circulation [66,67].

This regulatory limitation is primarily mediated by dipeptidyl peptidase-4 (DPP-4), a multifunctional enzyme responsible for the inactivation of GLP-1, GIP, and other bioactive peptides [68]. By attenuating incretin signalling, DPP-4 further constrains glucose regulation in obesity. Pharmacological inhibition of DPP-4 prolongs the activity of endogenous GLP-1, increases circulating levels of its active form, and sustains incretin receptor signalling without inducing significant adverse effects [69]. Consequently, the insulin–incretin–DPP-4 axis represents a central regulatory pathway in metabolic homeostasis and a key therapeutic target in obesity-related metabolic disorders [69].

Pro-inflammatory cytokines such as IL-6, IL-1β, TNF-α, IFN-γ, and MCP-1 contribute to the chronic low-grade inflammation characteristic of obesity by promoting metabolic dysfunction [70,71,72,73]. They are secreted by adipocytes and immune cells within adipose tissue under hypoxic conditions resulting from excessive adipose tissue expansion and inadequate blood supply [74]. Although anti-inflammatory cytokines such as IL-10, IL-4, and IL-13 may counterbalance this response, pro-inflammatory signalling remains predominant [75]. These molecules interfere with insulin signalling, contributing to insulin resistance, impaired glucose metabolism, and other obesity-related disorders, including cardiovascular disease [76]. High levels of IL-6 lead to increased synthesis and secretion of C-reactive protein by the liver [76].

C-reactive protein is a well-established biomarker of inflammation and is often elevated in individuals with obesity. Studies have shown a positive correlation between CRP levels and BMI, including in young adults [77]. In addition, CRP is considered a potential risk marker for other chronic inflammatory conditions, such as cardiovascular disease [78].

Lipoproteins such as LDL and oxidised LDL (ox-LDL) are elevated in obesity-related chronic inflammation. LDL-C is a well-established causal and therapeutically modifiable risk factor for atherosclerotic cardiovascular disease. Current evidence supports the cardiovascular benefits of achieving very low LDL-C levels without confirmed risks of cancer or neurocognitive disease [79]. In contrast, ox-LDL is better regarded as a potential biomarker and pathophysiological mediator of oxidative stress, vascular inflammation, and macrophage activation. Together with prostaglandin E2 (PGE2), ox-LDL may promote immune-cell differentiation into macrophages with pro-inflammatory profiles and contribute to obesity-related inflammation, type 2 diabetes, cancer, and cardiovascular disease [80,81].

Other potentially relevant biomarkers include sex hormones. Low levels of testosterone and sex hormone-binding globulin in men and high androgen levels in women have been associated with the development of obesity [82,83,84].

The aforementioned molecules, together with other circulating molecules, are increasingly being explored as potential biomarkers for obesity and its associated health risks [85]. Their clinical utility ranges from disease diagnosis and monitoring to predicting treatment response and evaluating therapeutic efficacy [86,87]. At the same time, these molecules represent a major focus of several omics disciplines aimed at personalising obesity management from both diagnostic and therapeutic perspectives [88].

In this context, obesity-related biomolecules can be classified into distinct biomarker categories such as diagnostic, prognostic, monitoring, pharmacodynamic/response, predictive, safety, and susceptibility/risk biomarkers (Table 2) [86].

## 5. Obesity: How Do We Treat It?

### 5.1. Behavioural Lifestyle Intervention

Modern obesity treatment usually begins with a clinically guided lifestyle intervention or behavioural lifestyle intervention (BLI). In terms of behavioural change, a variety of techniques have been described in Michie’s taxonomy [90]. The most commonly applied taxonomy groups in obesity treatment include goals and planning, feedback and monitoring, social support, shaping knowledge, natural consequences, and repetition alongside substitution [91].

Using these taxonomy groups and behavioural techniques, clinicians can develop specialised dietary and physical-activity programmes to evaluate and guide patients, either individually or in groups, towards achieving the desired weight-loss goals.

Among the dietary recommendations most frequently discussed in the scientific literature are hypocaloric regimens, including 500–600 kcal/day for 2 days/week or 5 days/week; higher-protein or higher-carbohydrate diets; the Mediterranean diet; the Palaeolithic diet, which excludes dairy products, salt, refined sugar, and grains while increasing the intake of vegetables, lean meat, fish, and nuts; and dairy-based regimens centred on milk, lean cheese, and yoghurt [92].

In terms of efficacy, Watson et al. [93] showed that energy-restricted dietary patterns combined with moderate physical exercise, as part of a holistic lifestyle modification, achieved similar weight loss, improvements in glycaemic control, and reductions in key cardiometabolic risk factors.

A systematic review showed that, among the 42 studies included in the meta-analysis, only 25 reported positive results in terms of BMI reduction [91]. Another systematic review published in 2023 analysed 324 articles investigating the combined impact of dietary changes and physical activity in patients with obesity and concluded that a hypocaloric diet combined with at least 175 min/week of endurance and strength-training exercise is recommended for effective weight management [94]. This approach may also reduce BMI and improve metabolic parameters such as the lipid profile and blood glucose [94].

### 5.2. Pharmacological Treatment

To enhance the effects of standard BLI, obesity treatment may include pharmacological therapy. In recent years, several agents grouped into three main classes have emerged: GLP-1 receptor agonists (semaglutide, tirzepatide, and liraglutide); malabsorptive medication (orlistat); and centrally acting medication (phentermine, phentermine/topiramate, bupropion, and setmelanotide). These agents exploit physiological mechanisms involved in appetite regulation, satiety, glucose metabolism, and body-weight control, offering an effective non-surgical alternative to conventional strategies. Major limitations include heterogeneous treatment responses and a substantial burden of adverse effects [95].

#### 5.2.1. GLP-1 Receptor Agonists

GLP-1 receptor agonists are among the most widely used pharmacological agents for obesity treatment, and their clinical use has increased substantially. Semaglutide exerts its effects by mimicking endogenous GLP-1 and activating the GLP-1 receptor. It thereby enhances glucose-dependent insulin secretion, suppresses glucagon release, delays gastric emptying, and promotes satiety [16]. Through these complementary mechanisms, semaglutide reduces energy intake and induces clinically meaningful weight loss.

In contrast, tirzepatide acts as a dual agonist of both the GLP-1 and GIP receptors. This combined incretin activity enhances its metabolic effects and has been associated with substantial improvements in glycaemic control and greater reductions in body weight compared with GLP-1 receptor agonist monotherapy in clinical trials [96].

Semaglutide efficacy was evaluated in the SELECT trial, which demonstrated that semaglutide induced an average weight reduction of approximately 10%, along with significant decreases in WC and WHtR [97].

The STEP 5 study further confirmed the ability of semaglutide to achieve sustained, clinically meaningful weight loss with an acceptable safety profile; gastrointestinal events were the most common adverse effects and were generally mild to moderate in severity. A recent meta-analysis reinforced these findings, showing that semaglutide 2.4 mg once weekly not only promotes substantial weight reduction but also improves blood pressure, glycaemia, inflammatory markers, and lipid parameters [98]. Real-world data also indicate that average weight loss reaches approximately 6% at three months and 11% at six months of treatment [99].

Specifically, the clinical efficacy of these agents may be influenced by target-gene variants—such as GLP1R (rs10305420) [100] and GIPR (rs10423928) [101,102], which dictate weight-loss velocity and adverse event susceptibility—as well as metabolic phenotypes such as low endogenous GLP-1 secretion, in which patients may act as “super-responders” and achieve accelerated weight loss. By mapping these genotypic variations and disease-comorbidity phenotypes (such as cardio-renal versus sleep-apnoea profiles), clinicians may be better able to match patients to the metabolic efficacy of tirzepatide or the established cardioprotective effects of semaglutide, shifting the paradigm from rigid body-mass metrics towards more precise biomolecular targeting [103].

Both semaglutide and tirzepatide are associated with gastrointestinal adverse effects such as nausea, vomiting, diarrhoea, and constipation [104]. These symptoms are often associated with treatment initiation or dose escalation and are usually transient. In the STEP 5 study, gastrointestinal events occurred in over 80% of patients receiving semaglutide [105]. For tirzepatide, treatment discontinuation due to gastrointestinal adverse effects was reported in 4–7% of participants, depending on the dose [106]. Importantly, discontinuation of either therapy often results in weight regain, highlighting the need for long-term maintenance and monitoring.

The use of these pharmacological agents is associated with improved glycaemic control and substantial reductions in body weight, accompanied by improvements in blood pressure and other cardiometabolic risk factors, thereby contributing to an overall reduction in cardiovascular risk [107,108].

Liraglutide is a long-acting GLP-1 receptor agonist engineered through structural modification of the native GLP-1 molecule, including the addition of a fatty acid side chain that promotes reversible binding to albumin. This modification prolongs its circulating half-life and enables once-daily administration. Evidence from 5 randomized controlled trials in individuals with obesity demonstrated that liraglutide treatment was associated with an additional weight reduction of approximately 4.4 to 6.1 kg compared with placebo [95].

To evaluate the efficacy and tolerability of incretin-based therapies, pharmacogenomic approaches may provide additional information on the substantial interindividual variability in treatment response. In a large genome-wide association study including 27,885 individuals treated with GLP-1-based medications, including semaglutide and tirzepatide, Su et al. investigated genetic determinants of self-reported weight loss and treatment-related adverse effects. A missense variant in GLP1R (rs10305420) was significantly associated with greater treatment efficacy, with each copy of the effect allele corresponding to an additional 0.76 kg of weight loss. Genetic variation within GLP1R was also associated with treatment-related nausea and vomiting. Furthermore, among individuals treated with tirzepatide, a dual GLP-1/GIP receptor agonist, variation within GIPR was specifically associated with gastrointestinal adverse effects, particularly nausea and vomiting. By integrating genetic findings with demographic and clinical characteristics, the authors stratified individuals according to both weight-loss response and risk of adverse effects. These findings highlight the potential of pharmacogenomic profiling for the future personalisation of obesity treatment [100].

#### 5.2.2. Centrally Acting Medication

##### Phentermine

Phentermine is a centrally acting sympathomimetic agent with stimulant properties related to those of amphetamines. Its anorexigenic effect is primarily mediated through enhancement of norepinephrine and dopamine, with additional effects on serotonin pathways, resulting in reduced appetite and food intake [109]. Owing to its sympathomimetic activity, treatment may be associated with adverse effects such as insomnia, palpitations, increased blood pressure, and gastrointestinal symptoms, and its indication is limited to patients with arterial hypertension. Clinical studies have reported mean body-weight reductions of approximately 7–8 kg following 12 weeks of phentermine therapy, and it is usually indicated for short-term treatment [110].

##### Phentermine/Topiramate

Topiramate is an anticonvulsant medication that is also widely used for migraine prophylaxis and has been associated with weight reductions of approximately 6–8 kg when administered as monotherapy [111]. Although the mechanisms responsible for its effects on body weight are not fully understood, modulation of hypothalamic γ-aminobutyric acid (GABA) signalling and other central pathways involved in appetite regulation is believed to contribute [112].

Combining topiramate with phentermine produces a greater weight-loss effect than either agent alone. In a randomised clinical trial, treatment with 15 mg phentermine/92 mg topiramate resulted in a mean body-weight reduction of 10.92% at 56 weeks, compared with 1.55% in the placebo group [113].

##### Naltrexone–Bupropion

Bupropion enhances dopaminergic and noradrenergic neurotransmission within hypothalamic circuits, thereby contributing to reductions in food intake. On the other hand, naltrexone, an opioid receptor antagonist originally used in the treatment of opioid and alcohol dependence, modulates reward-related pathways that may influence eating behaviour [114,115,116].

Clinical trials have demonstrated that naltrexone–bupropion treatment can produce total body-weight loss of up to approximately 6.1% after 56 weeks, compared with 1.3% in the placebo group. When combined with an intensive lifestyle intervention, mean weight reduction reached approximately 9.3%, compared with 5.1% in participants receiving placebo plus lifestyle therapy [115,116,117].

The most frequently reported adverse effects include nausea, headache, constipation, dizziness, vomiting, and dry mouth. In addition to weight loss, naltrexone–bupropion may reduce measures of central adiposity and LDL-C. Its effects on reward and craving pathways have also led to interest in its potential usefulness in patients with binge-eating disorder, but treatment selection should remain individualized [95].

Omics-based approaches may also contribute to the personalisation of centrally acting anti-obesity pharmacotherapy. Genomic evidence is currently the most developed in this field. In a recent study integrating deep phenotyping with genetic data, a machine-learning-derived genetic risk score for calories to satiation (CTS_GRS) was proposed. Individuals characterised by high CTS or a high CTS_GRS achieved greater weight loss during treatment with phentermine–topiramate, suggesting that genetic characterisation of satiation-related traits may help identify patients more likely to respond to centrally acting pharmacotherapy [118].

#### 5.2.3. Anti-Absorptive Treatments

##### Orlistat

Orlistat is a gastrointestinal lipase inhibitor that inhibits gastric and pancreatic lipases, thereby reducing the hydrolysis of dietary triglycerides and limiting the intestinal absorption of free fatty acids. Across clinical studies, treatment with orlistat has been associated with a total body-weight reduction of approximately 2.8% to 4.8% [119].

In addition to its effects on body weight, orlistat has demonstrated favourable changes in several cardiometabolic parameters, including reductions in total cholesterol, LDL-C, fasting plasma glucose, and both systolic and diastolic blood pressure [88,89,90]. However, its clinical utility is often limited by gastrointestinal adverse effects directly related to impaired fat absorption, such as flatulence, faecal urgency, faecal incontinence, and steatorrhoea-related symptoms. Long-term use may also contribute to deficiencies in fat-soluble vitamins. These tolerability issues are associated with relatively high treatment-discontinuation rates and have contributed to the comparatively limited use of orlistat in contemporary clinical practice [95].

### 5.3. Metabolic Surgical Procedures

A more established treatment option is metabolic surgery, which currently represents a viable long-term therapeutic strategy. Nevertheless, a shift in its indications and use has been observed in recent years, mainly because of its invasiveness and the potential for serious complications [120].

The indications for metabolic and bariatric surgery (MBS) have evolved substantially in recent years, increasingly incorporating the presence and severity of metabolic syndrome and obesity-related comorbidities into clinical decision-making. Nevertheless, patient selection remains closely linked to BMI-based stratification. According to the current American Society for Metabolic and Bariatric Surgery/International Federation for the Surgery of Obesity and Metabolic Disorders (ASMBS/IFSO) guidelines, MBS should be considered in patients with class I obesity (BMI 30.0–34.9 kg/m^2^) when non-surgical approaches, including BLI and pharmacological therapy, fail to achieve substantial and sustained weight loss or adequate improvement in obesity-related comorbidities. The indication is particularly relevant in patients with clinically significant metabolic disease, such as T2DM. Moreover, accumulating evidence suggests that patients undergoing MBS at lower BMI values may have a greater likelihood of achieving a postoperative BMI below 25 kg/m^2^ [121].

For patients with class II obesity (BMI ≥35 kg/m^2^), MBS represents a well-established therapeutic indication, irrespective of the presence or severity of obesity-related comorbidities. In this population, surgery is associated with substantial and durable weight loss, improvement or remission of metabolic complications, and favourable long-term effects on overall health outcomes and survival. Furthermore, behavioural and lifestyle interventions alone are generally less effective in achieving sustained and clinically meaningful weight reduction as the severity of obesity increases. The updated ASMBS/IFSO guidelines therefore represent a major shift from previous, more restrictive eligibility criteria and emphasize the central role of metabolic surgery within a comprehensive, long-term strategy for the management of obesity and its associated metabolic complications [121].

**Figure 1:**
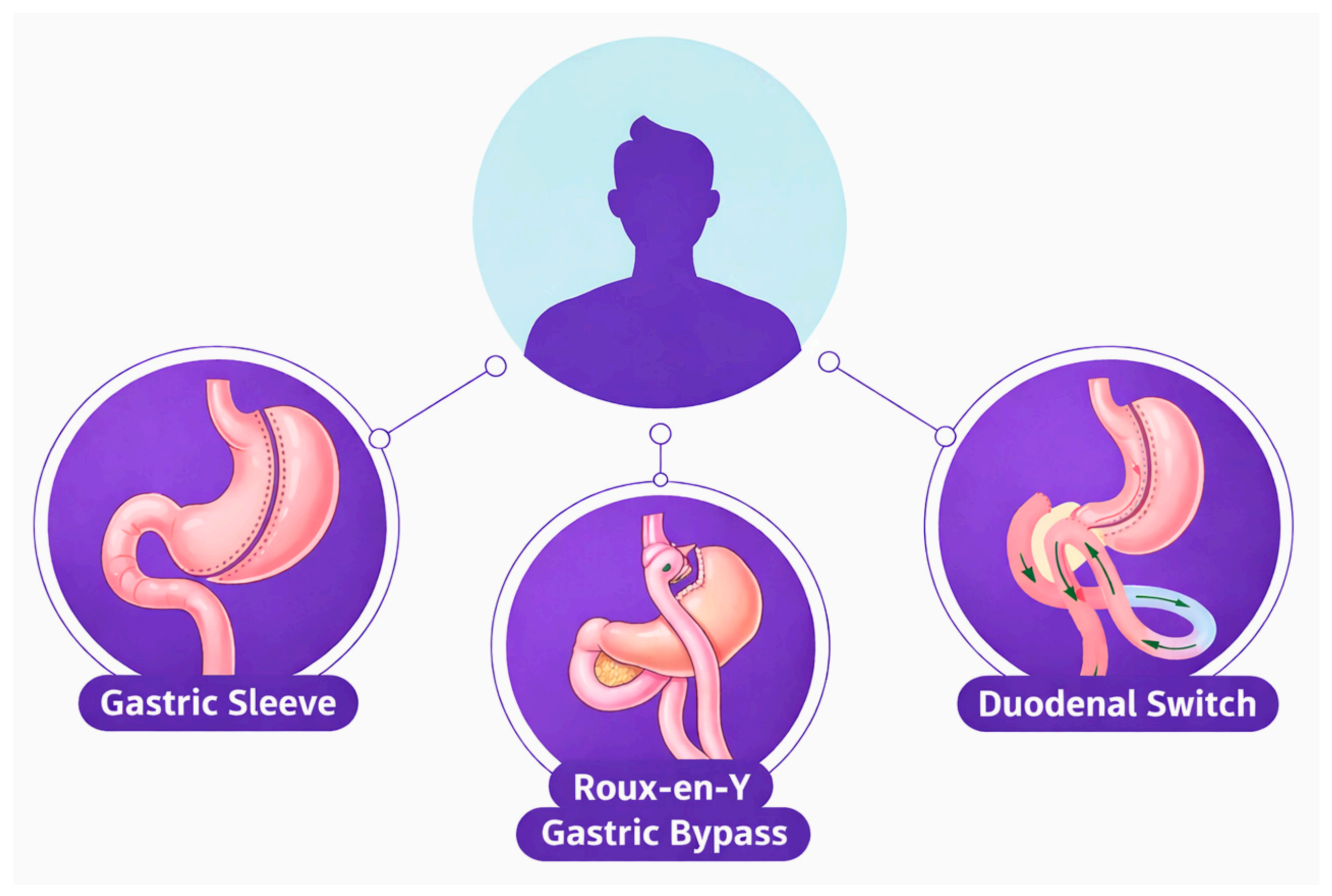
Main bariatric surgical procedures (This image was created using ChatGPT-5.6 Thinking and Adobe Photoshop CC 2019).

Bariatric procedures involve reconfiguration of the gastrointestinal tract anatomy (Figure 1), leading to reduced food intake and, for some procedures, reduced nutrient absorption. These are among the initial mechanisms through which weight loss occurs. However, the long-term effects of bariatric surgery are also mediated by postoperative alterations in metabolic and hormonal mechanisms that regulate hunger and satiety. Various molecules, peptides, and hormones contribute to postoperative changes in basal metabolism, appetite regulation, and gastric function. These changes may contribute to resetting body-weight regulation and maintaining long-term weight loss [122,123].

The efficacy of bariatric surgery has been evaluated in several long-term follow-up studies. One such study, the SLEEVEPASS trial, with 10 years of follow-up, showed that estimated weight loss (EWL) at 10 years was 43.5% after laparoscopic gastric sleeve (LGS) and 50.7% after laparoscopic Roux-en-Y gastric bypass (LRYGB). The study evaluated 240 patients, of whom 121 underwent LGS and 119 underwent LRYGB, with a baseline BMI of 45.9. The study also showed no statistically significant differences in remission rates for type 2 diabetes, obstructive sleep apnoea, or dyslipidaemia between the two surgical interventions [124].

A similar study, the SM-BOSS trial, evaluated percentage excess body mass index loss (%EBMIL) over a 10-year period. It included 217 patients randomised to undergo LSG or RYGB, of whom 156 were women. Approximately 65% of these patients completed the 10-year follow-up, at which point %EBMIL was 60.6% after LSG and 65.2% after RYGB. However, the mean percentage of total weight loss did not differ significantly between the two groups [125].

The overall surgical complication rate is approximately 2% after sleeve gastrectomy and 4–5% after gastric bypass [126,127].

Biliopancreatic diversion is one of the most invasive bariatric procedures, being reserved for cases of severe obesity [128].

Long-term complications of all types of bariatric surgery include vitamin B12 deficiency, iron deficiency, calcium and vitamin D malabsorption, impaired protein digestion, and reduced absorption of fat-soluble vitamins [129].

Given the invasiveness and potential complications of conventional metabolic surgery, together with the continuing development of the field, several “treatment-gap” options are also available, with variable rates of efficacy.

Laparoscopic gastric plication is a restrictive surgical procedure in which the stomach is folded inwards and sutured to reduce its size, similarly to LSG. Although the procedure can produce weight loss, significant gastric leaks and food intolerance have been reported, and long-term outcome data remain limited [130].

Endoscopic sleeve gastroplasty is an endoscopic counterpart to laparoscopic gastric plication. This procedure involves plicating the gastric wall from within the stomach using advanced endoscopic suturing devices that facilitate full-thickness suturing. By folding the gastric wall, gastric volume is significantly reduced, thereby restricting food intake [123,131].

Endoscopic gastroplasty offers an alternative to laparoscopic sleeve gastrectomy for patients who are unsuitable for surgery and may improve obesity-related comorbidities. Although it achieves a lower percentage of excess weight loss than its surgical counterpart, its efficacy and reduced invasiveness make it an acceptable intervention [132,133].

A widely used non-surgical procedure is the intragastric balloon, which involves introducing a balloon into the stomach. The balloon is inflated with 500 mL of saline solution to reduce overall gastric volume [123]. Depending on the type of balloon used, the procedure may require endoscopic insertion and subsequent removal. However, some commercially available balloons can be introduced in an outpatient setting by swallowing a capsule that is then inflated with saline, with intragastric placement confirmed by plain abdominal radiography. These balloons are excreted naturally after approximately four months [134].

This procedure has been shown to be effective for short-term weight loss, with most patients losing 12–15% of total body weight within the first year. However, more than half of patients who undergo this procedure subsequently regain weight and require further bariatric treatment [135,136].

Endovascular bariatric surgery (EBS) has been proposed as a minimally invasive technique that targets the vascular supply of the gastric fundus mucosa. This procedure reduces ghrelin secretion and regulates appetite [129].

The first human clinical trial evaluating this technique enrolled 40 patients (BMI 35.0–55.0 kg/m^2^; age 21–60 years) [137]. Participants were randomised 1:1 to a control group, which received intravenous sedation, or a study group, which underwent transcatheter bariatric embolotherapy (TBE) targeting the left gastric artery. Lifestyle support, including dietary and behavioural education for weight loss, was provided to both groups. Re-evaluation was performed after six months, and total body-weight loss was greater with TBE. In the intention-to-treat and per-protocol populations, weight loss was 7.4 kg/6.4% and 9.4 kg/8.3%, respectively, compared with 3.0 kg/2.8% and 1.9 kg/1.8% in the control group. As part of the peri-interventional assessment, all patients underwent gastroscopic evaluation after one week, and five cases of gastric ulceration were identified in the treatment group. These were treated with proton-pump inhibitors, with complete remission.

TBE does not yet have a standardised protocol, although a broadly similar approach is used in most studies. To identify the left gastric artery arising from the coeliac trunk, digital subtraction angiography is performed via femoral or radial arterial access. A decision to embolise the left gastroepiploic artery may then be made according to its contribution to gastric fundus vascularisation. Once the target vessel has been selected, selective cannulation and angiography are performed using the same catheter. Embolic materials may include microspheres of various sizes or polyvinyl alcohol (PVA) particles [129].

In a longitudinal study of 104 patients undergoing sleeve gastrectomy or Roux-en-Y gastric bypass, metabolomic and lipidomic profiling was performed before surgery and at 3 and 12 months postoperatively. Bariatric surgery was associated with significant changes in amino acid and lipid metabolism, including reductions in branched-chain amino acids and several triglyceride species, together with increases in phosphatidylcholines and phosphatidylethanolamines. These molecular changes paralleled improvements in metabolic health following surgery and provided additional information on postoperative metabolic adaptation beyond weight loss alone. Notably, Roux-en-Y gastric bypass induced greater changes in bile acid metabolism than sleeve gastrectomy, highlighting procedure-specific metabolic responses [138].

## 6. Personalisation in Obesity Management: A New Paradigm

**Figure 2:**
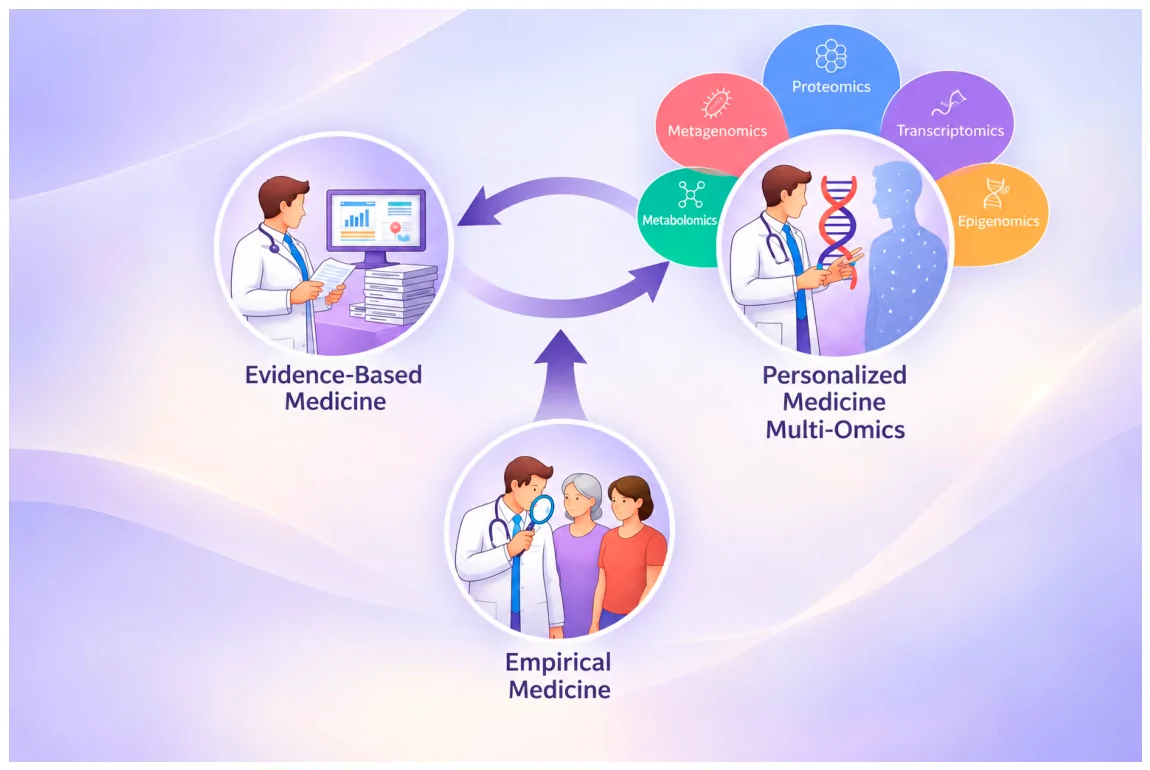
The shift in the medical paradigm (This image was created using ChatGPT-5.6 Thinking and Adobe Photoshop CC 2019).

Clinical evidence-based medicine (EBM) depends closely on practitioner judgement and the availability of data, which may not always be sufficient because of limited standardisation in diagnosis, stratification, classification, and individual therapeutic assessment. In this context, a more personalised approach is needed (Figure 2). Such an approach should be based on a more complex perspective integrating data acquired through different molecular tools capable of identifying distinct phenotypes and endotypes [139]. Nevertheless, EBM remains a fundamental paradigm in medicine and the core framework for clinical decision-making.

Individuals with obesity exhibit substantial heterogeneity in their responses to different diagnostic and therapeutic approaches, as well as in their preferences, behavioural habits, psychological profiles, and lifestyle patterns, which may contribute to limited long-term outcomes.

The imbalance between energy intake and energy expenditure represents a central element in the phenotyping and stratification of patients [140].

For example, a stratification approach proposed by A. Acosta identified correlations between quantitative traits of gastric function, such as satiation, and higher BMI. These traits can identify distinct obesity phenotypes, and a simple measurement of satiety may predict short-term response to obesity pharmacotherapy [140]. Compared with controls without obesity, patients with obesity show significant differences in quantitative gastrointestinal traits, including lower satiation, reflected by a higher volume intake required to feel full; accelerated gastric emptying of liquids and solids; and increased fasting gastric volume. In addition, a higher caloric intake was required to achieve satiety in individuals with abnormal WC [140].

Based on these observations, four phenotypes have been identified: abnormal satiation associated with abnormal hypothalamic brain perfusion (“hungry brain”); abnormal satiety associated with intestinal enteroendocrine cell dysfunction (“hungry gut”); abnormal emotional eating characterised by uncontrolled hedonic eating and associated with abnormal nucleus accumbens perfusion (“emotional eating”); and decreased resting energy expenditure evaluated by indirect calorimetry (“slow burn”) [140,141].

Although clinical stratification alone provides a valuable framework for improving the diagnosis and management of obesity, recent research has increasingly focused on a more holistic approach.

### 6.1. The Multi-Omics Landscape Driving Personalized Obesity Management

Whereas obesity research has historically relied largely on empirical analyses, a molecular perspective on the architecture of this disease is increasingly needed and is supported by evidence from genetic variants, epigenetic markers, proteomic and metabolomic signatures, neuroendocrine signalling, and the microbiome [142].

Analytical methods provide valuable insights into the molecular mechanisms involved in the pathophysiology of obesity, enabling the identification of biomarkers that may support more accurate diagnosis, prognosis, and personalised therapeutic strategies.

The hybrid approach to quantifying changes occurring in the pathophysiological context of obesity encompasses a complex technical and experimental arsenal, the main components of which include molecular biology techniques (PCR, gene sequencing, genotyping, microarray analysis, DNA methylation analysis, immunoassays, flow cytometry, chemiluminescence, and immunoprecipitation), biochemical techniques (spectrophotometry and enzymatic assays), and analytical techniques (mass spectrometry, chromatography, and nuclear magnetic resonance (NMR) spectroscopy) [143].

The aforementioned methods represent the starting point for a multi-omic approach to obesity. Correlating the multimolecular landscape with anthropometric indices widely used in current clinical practice is becoming an increasingly attainable objective. This may provide a basis for improving the long-term assessment of patients with obesity as understanding of the complex pathophysiology of the disease continues to expand.

The multi-omic strategy encompasses genotyping (genomics), dynamic molecular layers (epigenomics and transcriptomics), and phenotyping layers (proteomics, metabolomics, and microbiomics) [26,144].

From a genetic perspective, non-syndromic monogenic obesity accounts for only approximately 5% of cases worldwide, whereas polygenic obesity represents the vast majority. For the latter, a clear pattern of familial inheritance is more difficult to establish [145].

Genome-wide linkage analyses (GWLA) have proved effective in identifying genetic variants associated with monogenic forms of obesity. However, their impact on the identification of genetic markers in polygenic obesity is constrained by small cohorts, leading to limited reproducibility [146,147].

In contrast, genome-wide association studies (GWAS) are research tools based on advances in molecular biology that have led to the identification of multiple genetic loci associated with human obesity, including the fat mass and obesity-associated gene (FTO) [148] and the melanocortin 4 receptor gene (MC4R) [149]. In this way, multiple variants related to obesity and their phenotypic parameters, such as BMI, have been identified, including single-nucleotide polymorphisms [146,150,151].

Overall, both methods can identify links among different genotypes, phenotypes, and endotypes. In particular, GWAS conducted in large cohorts, such as the Genetic Investigation of ANthropometric Traits (GIANT), have identified numerous obesity-associated loci. Despite the discovery of hundreds of loci associated with obesity, together they account for only approximately 6% of BMI variance [146]. This finding underlines the heterogeneity of obesity and the need for continued research.

MC4R is a key component of the leptin–melanocortin pathway, which plays an important role in the regulation of energy homeostasis and obesity-related metabolism. Mutations affecting this pathway are typically associated with severe, early-onset obesity and are most commonly represented by pro-opiomelanocortin (POMC) deficiency and leptin receptor (LEPR) deficiency. Two multicentre studies evaluated the efficacy of setmelanotide specifically in these two conditions, demonstrating a weight loss of at least 10% in 80% of participants with POMC deficiency and in 45% of those with LEPR deficiency after one year of treatment [142].

A GWAS analysis using biobank data from multiple countries and integrating BMI and T2DM information identified biologically plausible mutations in candidate genes that could support the development of polygenic scores (PGS). The analysis showed that, among patients undergoing MS, a higher genetically predicted BMI was associated with a greater tendency towards postoperative weight regain, suggesting that additional interventions such as BLI may be beneficial. In contrast, no comparable association was observed for response to GLP-1 receptor agonist (GLP-1RA) therapy [152].

In this context, genetic testing, particularly through the use of polygenic risk scores (PGSs), may represent a valuable tool in the preoperative evaluation of patients [153].

The endeavour to personalise the medical approach to obesity involves not only large-scale population genetic studies but also clinical and molecular correlations. The ultimate goal is to identify the most appropriate treatment for each patient or specific patient group and to anticipate disease and treatment trajectories.

Obesity-related phenotypes are influenced by epigenetic mechanisms that integrate genetic susceptibility with nutritional, inflammatory, endocrine and environmental exposures.

Several epigenetic mechanisms and markers mediate obesity dynamics, including DNA methylation, histone acetylation, and chromatin remodelling [154,155,156]. These mechanisms regulate chromatin accessibility and the transcription of genes involved in adipogenesis, appetite control, insulin signalling, inflammation, mitochondrial metabolism, and energy expenditure [157]. An epigenome-wide association study (EWAS) conducted within the REGICOR framework identified and validated DNA methylation at 94 CpG sites associated with BMI and 49 CpG sites associated with WC [158]. Furthermore, more than 100 additional CpG sites were identified as being associated with BMI and WC [158].

Coding RNAs (including messenger RNAs) and non-coding RNAs, including microRNAs, long non-coding RNAs, and circular RNAs, provide significant information regarding cell function under different conditions [158]. A five-year integrative review synthesising transcriptomic studies on human tissues and cell lines from 2020 to 2025 emphasised the consistent involvement of immune-inflammatory activation, dysregulation of lipid and energy metabolism, and post-transcriptional regulation [159]. A 2025 omental adipose tissue transcriptomic study identified almost 3000 non-coding RNAs differentially expressed in obesity and correlated with lipid metabolic processes [160]. A more recent study combined transcriptomics with machine-learning algorithms, enabling discrimination between persistent and reversible obesity phenotypes based on distinct circulating microRNA panels [161].

The study of the proteome and metabolome in individuals with obesity using mass-spectrometry techniques contributes to a more complete pathophysiological picture of this metabolic disorder. Although these methods are costly and complex, they have become increasingly recognised in obesity research, as demonstrated by a growing number of relevant studies.

In one study, adipose tissue samples from 10 individuals with obesity and 10 controls were analysed using proteomic and metabolomic techniques [162]. Proteomic profiling identified and quantified 135 differentially expressed proteins (57 upregulated and 78 downregulated), with PHACTR2 and PLIN2 upregulated and ADAR downregulated in obesity. Metabolomic analysis identified 191 differential metabolites (110 upregulated and 81 downregulated), including exogenous compounds such as 4-vinylcyclohexene and asparagine-beta-xanthin, reflecting environmental and dietary differences between the cohorts, respectively.

Mass spectrometry has also been applied to obesity research through the analysis of plasma samples obtained from a cohort of 49 volunteers who were categorised according to BMI and MetS status. The study identified 231 proteins and 77 metabolites, among which DMBT1, Vanin-1, PTPRJ, β-hydroxybutyrate, α-tocopherol, and 5-oxoproline emerged as potential key molecules associated with obesity and MetS. Moreover, a dynamic workflow was successfully implemented in which variations in the concentrations of these molecules could be correlated with parameters currently used in clinical practice, such as BMI, HOMA-IR, and HOMA-β [163].

The oral microbiome exerts a complex and pleiotropic influence on the dynamics of gastrointestinal processes, and vice versa. This bidirectional interaction is strongly supported by the extensive exchange of metabolites and immune-signalling mechanisms between the two compartments [164].

Within the pathophysiological context of obesity, an unfavourable dietary and oral-hygiene regimen may trigger a cascade of interconnected changes. These include oral dysbiosis, characterised by the rapid expansion of pathogenic microbial populations such as *Porphyromonas gingivalis*, *Streptococcus parasanguinis, Actinomyces oris*, and *Oribacterium sinus*, followed by their translocation to the intestinal tract [165]. This process may compromise intestinal barrier integrity and promote both local and systemic inflammatory responses mediated, at least in part, by short-chain fatty acids and bile acids. Concomitantly, alterations in taste perception and appetite regulation may occur through the oral–gastrointestinal–brain axis [166].

In this regard, several studies have highlighted the potential contribution of the aforementioned mechanisms to the development of obesity-related alterations, thereby emphasizing the need for further investigation [166,167,168,169,170]. This emerging field provides the scientific community with an opportunity to explore a dynamic and still insufficiently characterized area, potentially contributing to a more comprehensive understanding of the complex mechanisms underlying obesity.

In a recent study conducted by Alonso-Allende et al. [169] in a cohort of 295 Spanish adults, a novel composite index (OBIndex) was implemented to provide a more refined classification of the investigated population based on the molecular fingerprint of the gastrointestinal microbiome, dividing participants into two subgroups according to lower or higher degrees of obesity. This strategy was designed to overcome the limitations associated with the use of individual established indices such as BMI, fat mass, and WC. Data were collected from both subgroups following an intervention aimed at reducing body mass and involving two distinct hypocaloric dietary regimens: a moderately high-protein (MHP) diet and a low-fat (LF) diet. The results enabled a more accurate classification of obesity status and led to the identification of four bacterial species with strong synergistic associations with the condition under investigation. Specifically, *Mediterraneibacter* and *Agathobacter* were associated with more severe obesity, whereas *Pseudobutyricicoccus* and *Intestinimonas* exhibited complementary activity to that of the former two species.

The current scientific literature shows a growing trend towards the implementation of multi-omics approaches to achieve a more comprehensive understanding of the biochemical processes underlying obesity. Whether referring to genomics, epigenomics, transcriptomics, proteomics, metabolomics, or metagenomics, these fields, although methodologically distinct, increasingly form an integrated framework with promising translational and clinical potential.

In this context, a structured overview of the available studies is warranted, and the main findings from the relevant literature are summarised in Table 3.

The findings of the studies presented above highlight the importance of effective patient stratification based on specific genetic, molecular, and microbiota-related signatures. Moreover, by integrating data derived from different omics domains, clinicians may tailor obesity treatment—including pharmacological, surgical, and interventional approaches—to distinct genotypes, phenotypes, and, particularly, endotypes, with the aim of improving therapeutic outcomes. Furthermore, the integration of machine learning (ML) into multi-omics data analysis is an emerging trend that may enhance the precision and predictive value of personalised medicine.

At the same time, the integration of omics-based techniques into the study of obesity metabolism underscores the limitations of conventional anthropometric measurements while providing more detailed approaches to the diagnosis and characterisation of obesity.

Nonetheless, the application of multi-omics approaches to the study of basal metabolism and obesity is still in its early stages and continues to face several challenges. The results of multi-omics studies may vary depending on the analytical techniques and platforms used, sample types, the populations studied, and cohort size. Many of the investigated biomarkers may also exhibit variability according to physiological or pathological conditions, and their interpretation may be confounded by obesity-related comorbidities. Similarly, the gut microbiome may vary in response to diet, stress, or the administration of different medications, while microbiome signatures still require further validation regarding their long-term stability and clinical relevance. Artificial intelligence (AI) models used for data interpretation and integration are also still at an early stage of development, which may contribute to the limited reproducibility of omics-based models. More broadly, the applicability of omics-based approaches in obesity is limited by methodological inconsistencies in sample collection and processing, population heterogeneity, reproducibility concerns, and socioeconomic constraints, including high analytical costs that restrict sample sizes, as well as stringent data-protection and anonymisation requirements [88].

Despite these challenges, the integration of multi-omics studies consistently contributes to a better understanding of the pathophysiological mechanisms underlying obesity and creates new opportunities for the personalisation of its clinical management.

### 6.2. Alternative Cost-Effective Techniques

An ideal detection and evaluation tool should combine high analytical sensitivity and specificity with reproducibility, low cost, rapid turnaround time, minimal invasiveness, and safety for both the patient and the investigator. Recent technological advances have sought to optimise these characteristics while enabling broader implementation in routine clinical practice.

To overcome the limitations of conventional techniques, increasing attention has been directed towards nanotechnology- and microfluidics-based platforms. These technologies improve analytical sensitivity and specificity while reducing sample-volume requirements, shortening assay time, and enabling miniaturised point-of-care diagnostics. Additional advantages include the elimination of multiple protocol steps, enhanced portability, and the potential for multiplex detection. Collectively, these benefits hold significant promise for transforming traditional clinical and laboratory workflows into faster and more accessible platforms.

Recent technological advances have led to the development of numerous diagnostic platforms that streamline clinical workflows and improve healthcare delivery. These platforms rely on various analytical approaches, including ultrasensitive vibrational spectroscopy.

Ultrasensitive vibrational spectroscopy techniques, including Raman spectroscopy, surface-enhanced Raman spectroscopy (SERS), and Fourier-transform infrared (FTIR) spectroscopy, have shown significant potential for the qualitative and quantitative assessment of molecular fingerprints in various pathologies, particularly through liquid and tissue biopsy analysis [180,181,182,183]. Raman spectroscopy is a vibrational spectroscopic technique based on the inelastic scattering of photons following monochromatic laser excitation. This technique enables the identification of molecules present in analysed samples through their molecular fingerprints. One major limitation is its low sensitivity when analysing biomolecules at low concentrations [184]. In principle, the sample is exposed to excitation laser light, which induces molecular vibration. The scattered photons may then lose or gain energy. The recorded spectra represent the energy difference between the incident photons and the inelastically scattered photons [185].

In the case of SERS, substrates with plasmonic properties are used to allow the amplification of the vibrational Raman spectra of analyte molecules. Spectra recorded with the help of these plasmonic substrates can support the development of new diagnostic strategies for different diseases within the broader field of liquid biopsy [185]. This method also enables the detection of analytes at low concentrations by amplifying local electromagnetic fields through the excitation of surface plasmons. In recent years, SERS has advanced substantially, including towards single-molecule detection. Its advantages include high sensitivity, real-time spectral analysis, and potential use in point-of-care applications [184].

FTIR spectroscopy is another analytical method based on the interaction of light with the sample. In this technique, infrared radiation interacts with molecules in the material at specific frequencies, inducing vibrational modes of the sample’s chemical bonds [186]. The method therefore reveals specific wavenumbers at which sample molecules absorb radiation, making it possible to identify analytes based on the bands present in the spectra. Spectroscopy-based methods therefore have the potential to meet several criteria of an effective diagnostic test simultaneously.

The integration of advanced analytical technologies has enabled the identification of disease-specific molecular signatures and biomarker patterns that correlate with clinical phenotypes and pathophysiological processes. Such molecular profiling supports more accurate diagnosis, risk stratification, and therapeutic decision-making, ultimately facilitating personalized interventions and improving patient outcomes.

Consequently, these advances are driving the development of highly sensitive, specific, rapid, and cost-effective diagnostic and screening strategies, an area that continues to attract considerable research interest worldwide.

## 7. Conclusions

Obesity is a complex disease that has evolved over recent decades into a global epidemic. Its complexity arises from the interplay of multiple socioeconomic, environmental, genetic, and medical factors, highlighting the substantial heterogeneity of obesity as a disease entity.

Obesity is closely associated with metabolic syndrome, diabetes mellitus, and a wide range of cardiovascular diseases, thereby exerting a major impact on the overall health status of the global population. These characteristics make the accurate diagnosis and characterization of obesity both highly important and particularly challenging.

Although anthropometric indices such as BMI and WC remain central to the diagnosis of obesity, a medical approach based solely on these measurements has major limitations. Such indices are unable to accurately capture the complex alterations in the genotype, phenotype, and endotype of patients with obesity.

Similarly, obesity treatment remains largely centred on anthropometric parameters, which restricts its adaptability to individual patient characteristics. Therapeutic options range from surgical interventions, which have demonstrated sustained efficacy over time but are associated with greater procedural risks, to pharmacological therapies such as semaglutide and tirzepatide. These molecules show considerable potential to transform the therapeutic approach to obesity; however, further studies are required to assess their long-term efficacy and clinical impact, as well as to support their adaptation and personalisation according to different obesity genotypes and phenotypes.

Given the marked heterogeneity of both the aetiology and clinical manifestations of obesity, the field has increasingly shifted towards a more personalised approach to patient management. This transition has been driven, among other factors, by advances in multi-omics technologies—including genomics, epigenomics, transcriptomics, metabolomics, proteomics, and metagenomics—as well as by the need for improved patient stratification. Accordingly, data generated through individual or integrated omics approaches may represent an important step towards the development of personalised strategies for the diagnosis and treatment of obesity.

In this context, adipose tissue is no longer regarded as a passive energy-storage compartment but rather as a metabolically active endocrine organ. It secretes a wide range of bioactive molecules, including hormones, chemokines, lipids, cytokines, proteins, and metabolites, many of which may contribute to improved patient stratification and provide potential targets for more individualised therapeutic interventions.

As a result of these investigations, several obesity-associated molecules have emerged as potential biomarkers, whereas the clinical relevance of others remains to be further validated. In addition, a number of techniques currently used primarily in research settings may demonstrate future potential for clinical application. Together, these approaches contribute to ongoing efforts to develop a more precise and personalised medical framework for the diagnosis, stratification, and treatment of obesity.

The personalised approach to obesity that characterises 21st-century medicine integrates anthropometric indices, clinical data, biomarkers, and advanced analytical methods with omics-based information. The aim of this integrative framework is to provide a more accurate characterisation of the pathophysiological alterations associated with obesity and its comorbidities, to stratify patient groups according to specific genomic, proteomic, metabolomic, and microbiome-related features, and to facilitate a more tailored therapeutic approach. It may also contribute to the assessment of established treatment options, the identification of novel therapeutic strategies, and the evaluation of both short- and long-term treatment responses in patients with obesity.

## Figures and Tables

**Table 1:** EOSS according to Canning et al. [24].

EOSS Stage	Characteristics	Clinical Manifestation	Paraclinical Evaluation
0	No apparent obesity-related risk factors, physical symptoms, psychopathology, functional limitations, or impairments of well-being.	No EOSS factors reported	No EOSS factors reported
1	Presence of subclinical risk factors, mild physical symptoms, mild psychopathology, mild functional limitations, or mild impairment of well-being.	No EOSS factors reported	Glucose ≥ 5.6 mmol/L Cholesterol ≥ 5.2 mmol/L Triglycerides ≥ 1.7 mmol/L HDL ≤ 1.6 mmol/L LDL ≥ 3.3 mmol/L SBP ≥ 130 mmHg
2	Presence of established obesity-related chronic disease, moderate limitations in daily activities, and/or moderate impairment of well-being.	Diagnosed type 2 diabetes or type 2 diabetes medication Diagnosed hypercholesterolemia Diagnosed hyperlipidaemia or hyperlipidaemia medication Diagnosed hypertension or hypertension medication Sleep apnoea Gout Arthritis Anxiety Atherosclerosis Fatty liver Congestive heart failure medication Blood thinner medication Depression	Glucose ≥ 6.9 mmol/L Cholesterol ≥ 6.2 mmol/L Triglycerides ≥ 2.2 mmol/L HDL ≤ 1.0 mmol/L LDL ≥ 4.1 mmol/L SBP ≥ 140 mmHg DBP ≥ 90 mmHg
3	Established end-organ damage, significant psychopathology, major functional limitations, and/or significant impairment of well-being.	Angina Heart attack Heart failure Thrombosis Coronary artery disease Chronic obstructive pulmonary disease Dyspnoea Exercise dyspnoea Coronary artery bypass surgery Stroke	No EOSS factors reported
4	Severe (potentially end-stage) disabilities from obesity-related chronic diseases, disabling psychopathology, severe functional limitations, and/or severe impairment of well-being.	No data on these factors available to evaluate this stage	No data on these factors available to evaluate this stage

SBP, systolic blood pressure; DBP, diastolic blood pressure.

**Table 2:** Key biomolecules involved in obesity and their clinical relevance according to [89].

Biomolecule	Main Source	Function	Expression Pattern in Obesity	Biomarker Category
Leptin	Adipose tissue	Satiety regulation	Increased; resistance	Diagnostic & monitoring
Ghrelin	Stomach	Hunger stimulation	Decreased; resistance	Diagnostic & Predictive
Adiponectin	Adipose tissue	Anti-inflammatory; insulin sensitizer	Decreased; resistance	Diagnostic
Resistin	Adipose tissue	Pro-inflammatory; insulin resistance; adipocyte proliferation and angiogenesis	Increased	Diagnostic, predictive & cardiovascular risk
Visfatin	Visceral adipose tissue and immune cells	Insulin-like; inflammatory	Increased	Insulin resistance & diagnostic
Omentin-1	Visceral adipose tissue	Insulin sensitizer; anti-inflammatory; anti-atherogenic	Decreased	Predictive & cardiovascular risk
Chemerin	Liver and adipose tissue	Adipocyte development and metabolic function; insulin regulation; low-grade inflammation	Increased	Insulin resistance & cardiovascular risk
Insulin	Pancreas	Cellular energy and macronutrient balance, satiety regulation	Increased; resistance	Predictive
DPP-4	Adipose tissue and immune cells	Catalyses GLP-1 degradation	Increased	Pharmacodynamic
GLP-1	Intestinal L-cells	Promotes insulin secretion and effects	Decreased	Pharmacodynamic
Pro-inflammatory cytokines	Adipocytes and immune cells within adipose tissue	Chronic inflammation; metabolic dysfunction	Increased	Inflammation & monitoring
C-reactive protein	Liver	Inflammatory	Increased	Inflammation & cardiovascular risk
LDL & ox-LDL	Bloodstream	Inflammatory	Increased	Cardiovascular risk & inflammation
Sex hormones	Suprarenal glands and gonads	Inflammatory	Modified	Obesity development

**Table 3:** Synthesis of Multi-Omics Integration in Obesity Research.

Title	Integrated Omics	Study Design	Intervention	Main Finding and Relevance
Multi-omic definition of metabolic obesity through adipose tissue–microbiome interactions [171]	Plasma metabolome Plasma proteome Genomics Faecal microbiome	1408 individuals; external validation cohort n = 466; participants without T2DM, aged 50–65 years; dietary intake evaluated using the MiniMealQ questionnaire; blood and stool samples collected. In the validation cohort (n = 466), both BMI and metabolome-informed BMI (metBMI) were calculated. Non-T2DM, 50–65 years old, Dietary intakes evaluated by MiniMealQ questionnaire, Blood samples and stool samples. Control cohort of 466, for which both BMI and metabolome-informed BMI (metBMI) were calculated	Metabolic surgery, lifestyle intervention	A correlation between conventional BMI and the metabolomic profile of the analysed cohort was proposed through the development of a metBMI, which accounted for approximately 52% of the variance in standard BMI. Higher metBMI values were associated with an increased prevalence of obesity-related comorbidities. An inverse association was also observed between metBMI and gut microbiome integrity, with higher metBMI values being associated with greater alterations in the intestinal microbiome.
Multi-omic modelling of body mass index response to dietary weight loss intervention [172]	Genomics Gut microbiome Microbial functional pathways Metabolomics	150 adults; 12-month, forecast longitudinal and BMI change (ΔBMI). Daily Caloric Restriction vs. Intermittent Fasting Trial (DRIFT), a 1-year randomized trial which compared weight loss generated by either Daily Caloric Restriction (DCR) or 4:3 Intermittent Fasting (IMF) in healthy adults (N = 165), aged 18–65 years, with overweight or obesity (BMI 27–46) Mixed Effects Random Forests (MERF) and GLMM-Lasso Genomic DNA from previously collected whole blood samples Serum metabolome was processed with NMR to infer concentrations of 250 metabolites, including lipids, lipoproteins, amino acids, and glycolysis-related metabolites Stool samples were collected for gut microbial taxonomy, microbial functional gene data collection, and gut microbial flux data	Behavioural weight-loss intervention	Integrated genomics, metabolomics and metagenomics in predictive statistics modelling to forecast BMI trajectories and change in BMI. Blood metabolomic data have shown improved prediction values beyond standard clinical variables, and the combined omics score explained a meaningful proportion of interindividual variability in BMI response.
GYG1 as a Dual Biomarker of GLP-1 Receptor Agonist Weight-Loss Response: Findings from an Integrative Multi-Omics Substudy of a Phase II Trial [173]	Proteomics Metabolomics Lipidomics	221 longitudinal plasma samples High-resolution mass spectrometry quantified molecular features clinical BMI trajectories Linear regression analysis identified longitudinal biomarkers	Pharmacological treatment with the GLP-1 receptor agonist GZR18 (bofanglutide);	Identification of BMI change biomarkers and evaluation of GZR18–bofanglutide treatment efficacy. Glycogenin 1 (GYG1) dual-function biomarker for GLP-1 therapy, merging baseline prediction and clinical treatment monitoring via multi-omics profiling.
Multi-omics Picture of Obesity in Young Adults [174]	Genomics Proteomics Metabolomics	101 fasted human plasma samples from young Caucasian adults (18–44 years old) with underweight to obesity; Anthropometric measurements: BMI Genomic DNA from blood samples Standard proteomic profiling also from blood samples Metabolomic analysis was conducted on low molecular weight plasma samples	No intervention	This study evaluates the capacity of proteomics, metabolomics and genomics to predict the BMI of young adults, with proteomic and metabolomic data being the most accurate in this endeavour. Adding genomic information did not further improve the model, thus demonstrating that more omics layers do not necessarily guarantee better prediction
Machine learning-based clustering identifies obesity subgroups with differential multi-omics profiles and metabolic patterns [175]	Proteomics Metabolomics	243 individuals with obesity Multi-Ethnic Study of Atherosclerosis (MESA) cohort BMI ≥ 30 kg/m^2^ Blood samples SOMAscan-generated proteomics Machine learning-based integrative unsupervised clustering to identify proteomics and metabolomics-defined subpopulations. IClusterBayes	No intervention	Using machine learning protocols and unsupervised clustering to integrate molecular data with clinical data, two obesity subgroups with different cardiometabolic profiles. The study supports molecular subphenotyping beyond BMI-based classification.
Multi-omics reveal unique signatures of human epiploic adipose tissue related to systemic insulin resistance [176]	DNA methylome Transcriptomics Proteomics	70 patients with metabolic surgery: insulin-sensitive (IS) and IR Control cohort–adipose tissue donors with controlled anthropometric features Samples: different types of adipose tissues (AT); mesenteric AT (mesAT)/epiploic AT (epiAT)/subcutaneous AT (scAT) Blood, immunohistochemistry and gene expression analyses Methylome analyses in a sub-cohort consisting of nine IS subjects and nine IR individuals. Proteome/Transcriptome analyses were performed in the same subcohort.	Roux-en-Y gastric bypass	Comparison between insulin-sensitive and insulin-resistant phenotypes in different types of adipose tissues Multi-omics signatures distinguish between IR and IS phenotypes despite BMI values. Epiploic adipose tissue showed particularly strong discriminatory power. Molecular tissue profiling can reveal clinically relevant metabolic heterogeneity hidden by conventional anthropometry.
Baseline Gut Metagenomic Functional Gene Signature Associated with Variable Weight Loss Responses following a Healthy Lifestyle Intervention in Humans [177]	Proteomics Metabolomics Metagenomics Genomics	105 participants over 18 years old divided into 3 distinct groups: weight loss (overweight (BMI > 25) or obese (BMI > 30)), no weight loss (BMI > 25) and normal weight (BMI < 25) Blood samples and stool samples 16S rRNA gene sequencing	Wellness program including healthy lifestyle	After intervention, the “weight loss” group showed an increase in adiponectin levels and decreases in APOE, C5, CRP, LBP, NEGR and PRSS3 levels, suggesting an improvement in metabolic and immunological status. Following proteomics, metabolomics and genomics, it was found that the association between BMI and diet or phenotype were largely independent of weight loss. On the other hand, metagenomics revealed 31 functional genes of the gut microbiome that may be related to weight loss, whereas the abundance of bacterial genera appeared to be less relevant. In addition, genes involved in protein catabolism, stress response, antibiotic peptide synthesis and respiration were found to be correlated with resistance to weight loss.
Multi-omic Predictors of Short-Term Weight Loss and Clinical Outcomes During a Behavioural-Based Weight Loss Intervention [178]	DNA methylation Metabolomics Gut microbiome	62 adults, 18–55 years, BMI 27–45 kg/m^2^ Identifying baseline omics predictors of changes in clinical outcomes at 3 and 6 months Whole-blood DNA, plasma metabolites, gut microbial–stool samples Multi-omic data were compared with 10 clinical outcomes that change over time, between evaluations	1-year behavioural weight loss intervention	Integrated models predicted interindividual changes in clinical outcomes like weight, waist circumference, triglycerides, glycolyzed haemoglobin, HOMA-IR, CRP, leptin, and ghrelin. These models provide a foundation for the development of more precise, patient-tailored BLI regimens.
Integrative phenotyping of glycaemic responders upon clinical weight loss using multi-omics [179]	Adipose-tissue transcriptomics Plasma proteomics and metabolomics	781 participants glycaemic responders vs. non-responders Multicentre, randomized controlled dietary intervention study, involving eight European countries Overweight/obese, non-diabetic adults, BMI between 27 and 45 kg/m^2^ Clinical variables: BMI, weight, body fat from bio-impedance and waist circumference Glycaemic control measures: fasting glucose/insulin; HOMA-IR (glucose (mmol/L) x insulin (µU/mL)/22.5); and Matsuda index (measure of insulin sensitivity derived from 2-h oral glucose tolerance tests). Total lipid levels (cholesterol, triacylglycerol (TAG), HDL and derived LDL). total free fatty acid (FFA)) were analysed by means of blood biochemistry. The Visceral Adiposity Index (VAI), a robust index of visceral fat, was derived using the formula proposed by Amato Abdominal scAT biopsies were obtained by needle aspiration under local anaesthesia Plasma omics analysis on blood samples	8-week weight-loss complete meal replacement LCD 800 kcal/day diet and a 26-week weight maintenance diet	Provides an integrated multi-omics model that distinguishes between individuals with improved glycaemic control and those with poor glycaemic control even after an important weight loss. This model improves the prediction of glycaemic control in LCD intervention.

## Data Availability

Data sharing is not applicable to this article as no datasets were generated or analyzed during the current study.

## Abbreviations

The following abbreviations are used in this manuscript:

WHO	World Health Organization
BMI	Body Mass Index
WC	Waist Circumference
WHtR	Waist-to-Height Ratio
WHR	Waist-to-Hip Ratio
EOSS	Edmonton Obesity Score System
MetS	Metabolic Syndrome
NHANES	U.S. National Health and Nutrition Examination Survey
DEXA	Dual-Energy X-Ray Absorptiometry
CDC	Centres For Disease Control and Prevention
IR	Insulin Resistance
LDL	Low-Density Lipoprotein
HDL	High-Density Lipoprotein
C	Cholesterol
CVD	Cardiovascular Disease
T2DM	Type 2 Diabetes Mellitus
CRP	C-Reactive Protein
IGT	Impaired Glucose Tolerance
DM	Diabetes Mellitus
EGIR	European Group for Study of Insulin Resistance
IFG	Impaired Fasting Glucose
IDF	International Federation for Diabetes
MASLD	Metabolic Dysfunction-Associated Steatosis Liver Disease
TNM	Tumour Nodes and Metastasis
NYHA	New York Heart Association Functional
SBP	Systolic Blood Pressure
DBP	Diastolic Blood Pressure
GLP-1	Glucagon-Like Peptide-1
GIP	Glucose-Dependent Insulinotropic Polypeptide
ox-LDL	Oxidized LDL
DPP-4	Dipeptidyl Peptidase-4
PGE2	Prostaglandin E2
BLI	Behaviour Lifestyle Intervention
GABA	Γ-Aminobutyric Acid
MBS	Metabolic and Bariatric Surgery
ASMBS/IFSO	American Society for Metabolic and Bariatric Surgery/International Federation for the Surgery of Obesity and Metabolic Disorders
EWL	Estimated Weight Loss
LGS	Laparoscopic Gastric Sleeve
LRYGB	Laparoscopic Roux-Y Gastric Bypass
CRS_GRS	Genetic Risk Score For Calories To Satiation
%EBMIL	Percentage Excess Body Mass Index
EBS	Endovascular Bariatric Surgery
TBE	Transcatheter Bariatric Embolotherapy
PVA	Polyvinyl Alcohol
EBM	Evidence-Based Medicine
NMR	Nuclear Magnetic Resonance
GWLA	Genome-Wide Linkage Analyses
GWAS	Genome-Wide Association Studies
FTO	Fat Mass and Obesity Gene
MC4R	Melanocortin 4 Receptor Gene
POMC	Pro-Opiomelanocortin
LEPR	Leptin Receptor
PGS	Polygenic Score
GLP-1RAs	GLP-1 Receptor Agonists
EWASs	Epigenome-Wide Association Studies
MHP	Moderately High-Protein
metBMI	Metabolome-Informed BMI
ΔBMI	BMI Change
DRIFT	Daily Caloric Restriction vs. Intermittent Fasting Trial
DCR	Daily Caloric Restriction
IMF	Intermittent Fasting
MERF	Mixed-Effects Random Forests
GYG1	Glycogenin 1
MESA	Multi-Ethnic Study of Atherosclerosis
IS	Insulin-Sensitive
AT	Adipose Tissue
mesAT	Mesenteric Adipose Tissue
epiAT	Epiploic Adipose Tissue
scAT	Subcutaneous Adipose Tissue
LCD	Low-Calorie Diet
TAG	Triacylglycerol
FFA	Free Fatty Acid
VAI	Visceral Adiposity Index
ML	Machine Learning
AI	Artificial Intelligence
SERS	Surface-Enhanced Raman Spectroscopy
FTIR	Fourier-Transform Infrared Spectroscopy
